# Lymph Node Assessment in Endometrial Cancer: Towards Personalized Medicine

**DOI:** 10.1155/2013/892465

**Published:** 2013-09-26

**Authors:** Fabien Vidal, Arash Rafii

**Affiliations:** ^1^Stem Cell and Microenvironment Laboratory, Weill Cornell Medical College in Qatar, Education City, Qatar Foundation, Doha, Qatar; ^2^Department of Genetic Medicine, Weill Cornell Medical College, New York, NY 10065, USA; ^3^Department of Genetic Medicine and Obstetrics and Gynecology, Weill Cornell Medical College, Stem Cell and Microenvironment Laboratory Weill Cornell Medical College in Qatar, Qatar Foundation, Doha 24144, Qatar

## Abstract

Endometrial cancer (EC) is the most common malignancy of the female reproductive tract and is increasing in incidence. Lymphovascular invasion and lymph node (LN) status are strong predictive factors of recurrence. Therefore, the determination of the nodal status of patients is mandatory to optimally tailor adjuvant therapies and reduce local and distant recurrences. Imaging modalities do not yet allow accurate lymph node staging; thus pelvic and aortic lymphadenectomies remain standard staging procedures. The clinical data accumulated recently allow us to define low- and high-risk patients based on pre- or peroperative findings that will allow the clinician to stratify the patients for their need of lymphadenectomies. More recently, several groups have been introducing sentinel node mapping with promising results as an alternative to complete lymphadenectomy. Finally, the use of peroperative algorithm for risk determination could improve patient's staging with a reduction of lymphadenectomy-related morbidity.

## 1. Introduction

Endometrial cancer (EC) is the most common malignancy of the female reproductive tract with an estimated 47.130 new cases in 2012 in the United States [1]. Most patients are diagnosed with an early-stage disease, and the overall survival for stage I is about 85–91% [2]. Nevertheless, patients with advanced disease and unfavorable pathological characteristics have a guarded prognosis [3]. The most significant prognostic factors are histological type and grade, depth of myometrial involvement, lymphovascular invasion, and lymph node (LN) status [4]. 20% of the patients with EC extending outside of the uterus (stages II and IIIA-B) and 10% of the patients with clinical stage I disease have LN metastases (LNM) [2]. Therefore, removal of pelvic and paraaortic LN has been recommended as part of a comprehensive surgical staging including total hysterectomy and bilateral salpingo-oophorectomy [2, 3]. 

The management of EC has always been heterogeneous across different institutions and countries, in particular regarding LN staging [5–8]. Recently, the publication of 2 randomized trials and 1 meta-analysis [9–11] increased controversy on LN assessment. Indeed, both trials demonstrated that pelvic lymphadenectomy did not improve disease free and overall survival rates, and therefore should not be recommended as routine procedure. However, several flaws in their design (no randomization for postoperative adjuvant therapy, no systematic paraaortic lymphadenectomy) make the strength of these conclusions questionable [12, 13]. Despite that the therapeutic value was only supported by retrospective studies, lymph node dissection (LND) is, to date, the only way to fully stage the disease and to determine patients that are likely to benefit from adjuvant therapy [12, 14–16]. Finally, there is still a lack of accurate imaging procedures determining the extent of extrauterine disease; USPIO-enhanced MRI might improve preoperative staging, allowing detection of metastases in normal-sized LN, but it needs more studies to be deemed as a useful and reliable technique [17, 18]. 

Altogether, several questions have not been clearly answered by previous studies. Do LNM impact prognosis? What is the optimal LN staging? Who are the patients benefiting more from LN staging? What are the alternatives to complete LND? The aim of this review is to describe the state of the art in LN assessment and to determine the current methods and indications for surgical LN staging. 

## 2. Lymph Node Metastasis and Prognosis

The following studies clearly demonstrate that LN metastasis is the most important prognosis factor in early-stage EC. Morrow et al. evaluated the correlation between pathologic risk factors and outcomes in clinical stages I and II. They showed that the 5-year disease-free survival (DFS) was 90% in patients without LNM, 75% in patients with pelvic LNM, and 38% with paraaortic LNM [19]. Lurain et al. reported similar findings, with a 5-year DFS of 54% in patients with nodal involvement, whereas it was computed at 90% in those without LNM [20]. They also described an overall recurrence rate of 48% with positive LN compared to 8% with negative LN (45% with positive pelvic LN and 64% with positive paraaortic LN).

 Among patients with LNM, paraaortic LN involvement undoubtedly portends a poorer prognosis [21, 22] and occult paraaortic nodal disease becomes a substantial concern [23]. In a recent retrospective study, Garg et al. underlined these findings [24]. Among 2559 stage IIIC EC patients, those presenting with paraaortic involvement were more likely to die from their diseases (HR = 1.40 CI). Thus, the FIGO modified its staging of EC and sorted stage IIIC into 2 subgroups according to the paraaortic LN status [25]. 

 The precise staging of the patients has, therefore, clinical relevance for optimizing further treatments. Adjuvant chemotherapy is essential for the treatment of stage III and IV EC [26]. A randomized trial compared whole abdominal radiotherapy to combined chemotherapy (cisplatin and doxorubicin) and showed the superiority of chemotherapy [27]. The 5-year survival was 53% in patients given chemotherapy compared to 42% in the radiation group. However, patients who received chemotherapy experienced more frequent and more severe acute toxicity. Multimodality therapy is also commonly used for women with advanced disease and combines the systemic effects of chemotherapy with the local control provided by radiation [28, 29]. In a multicenter retrospective analysis of patients stages III and IV EC, sequential CRC (chemotherapy followed by radiation and then further chemotherapy) was associated with improved DFS and OS compared to other sequential modalities [29]. 

 Further trials will determine the most accurate design for adjuvant chemotherapy and the ideal sequence for multimodality approaches, according to the FIGO stage. Nonetheless, the whole therapeutic sequence will be based on the identification of patients with advanced disease and particularly of those with stage IIIC EC. 

## 3. Standard Lymphadenectomy

The primordial role of lymph node staging to optimize adjuvant therapy mandates careful characterization of the lymphadenectomy procedures.

### 3.1. Technical Aspects

Currently, several methods are available to treat and stage patients presenting with EC. While the traditional approach through laparotomy can still be used, minimally invasive surgical techniques such as traditional and robotic-assisted laparoscopy should be preferred since they are equally efficacious in terms of overall and disease-free survivals and associated with reduced peroperative morbidity and hospital stay [30, 31]. Indeed, guidelines of the French society of Gynecologic oncology recommended in 2011 the initial use of laparoscopic approach for stage I EC [32]. Ballester et al. confirmed in a prospective multicenter study a higher rate of laparoscopy (78%) versus open surgery for the management of stages I and II EC [33].

### 3.2. Anatomical Landmarks

Pelvic lymphadenectomy includes complete skeletonization of the common, external, and internal iliac vessels and the harvesting of all fatty and lymphatic tissues above and below the obturator nerve. Even if isolated paraaortic involvement appears to be low (up to 6%), it occurs in approximately 50% of the patients with positive pelvic LN and is of great prognostic value [7, 34, 35]. Therefore, paraaortic area should be systematically part of the LND. Its anatomical landmarks should consider the presacral area as lower boundary, right ovarian vein insertion to vena cava as right upper border (right paraaortic side), and left renal vessels as left superior margin (left paraaortic side). Indeed, up to 77% of the patients with paraaortic involvement are found to have LNM above the level of the inferior mesenteric artery (high left paraaortic side) [34]. Among these patients, 40% presented with LNM in both low (below the inferior mesenteric artery) and high left paraaortic sides and 60% in high left side only [34]. However, according to Soliman et al., the anatomic borders of LND continue to be controversial. Most of gynaecological oncologists (50%) use the inferior mesenteric artery as upper boundary and only 11% carry on dissection to the level of renal vessels [8]. 

### 3.3. Defining the Number of Lymph Nodes

There is still no defined number of lymph nodes to be removed to ensure a reliable sampling, and the sensitivity of LND increases with the removal of more nodes. Indeed, the logistic regression model proposed by Chan et al. demonstrated that resection of 21 to 25 nodes provided an 80% probability of detecting at least 1 positive lymph node [36]. In the Mayo Clinic experience, Bakkum-Gamez et al. considered a diagnostic LND as adequate if it retrieved at least 22 pelvic and 10 paraaortic LNs [37]. Several retrospective studies suggest a correlation between the number of excised LN and clinical outcome. Cragun et al. reported a significant increase of overall and progression-free survivals in patients with poorly differentiated early stage EC with more than 11 pelvic nodes retrieved [15]. Lutman et al. defined a cut-off of 12 or more pelvic LNs for high-risk (HR) patients [38]. However, as demonstrated by Cormier et al., the LN count varies markedly from a pathologist to another, yielding to exercise caution when drawing conclusions from published LN counts in EC research [39]. Actually, the surgeon remains the only one to know whether the lymphadenectomy he performed was complete.

### 3.4. Therapeutic Value of Lymphadenectomy

The MRC ASTEC and Panici et al. studies reached similar conclusions, showing no benefit from pelvic LND in terms of overall survival and DFS [10, 11], but these studies appear to be seriously flawed [13]. Indeed, besides being underpowered, the design of the ASTEC trial negated any possible advantage to LNM detection by not giving adjuvant therapy to those patients with nodal involvement. In the Italian trial, postoperative radiation was not standardized and was delivered more often to patients who did not undergo LND. Finally, paraaortic LND was not systematically performed in both studies. Indeed, those 2 randomized trials focused on the therapeutic value of pelvic LND, when its main purpose is the detection of lymphatic spread and thus the identification of patients who might profit from adjuvant therapy.

Conversely, only retrospective data support the therapeutic role of LND. Chan et al. showed that LND was associated with improved 5-year disease-specific survival in patients with stage IB grade 3 and advanced diseases, whereas, no survival benefit was observed in low risk group [40]. Kilgore et al. evaluated survival in a cohort of 649 stages I and II patients and found that the LND group had an improved 5-year overall survival (90%) in comparison to patients that did not undergo lymphadenectomy [16]. More recently, the SEPAL study reported a significantly increased overall survival (HR 0.53) in patients undergoing pelvic and paraaortic lymphadenectomies versus pelvic LND alone [41]. Finally, Kim et al. analyzed a cohort of 257 patients presenting with intermediate risk (IR) or HR. Among these patients, 164 underwent a pelvic LND alone and 93 a LND comprising both pelvic and paraaortic areas. Whereas recurrence rate was similar in the 2 groups, the incidence of extrapelvic relapse was significantly higher in the pelvic LND group, suggesting a therapeutic benefit from paraaortic LND.

### 3.5. Side Effects

Whereas it constitutes a longstanding argument against LND, the rate of complications from lymphadenectomy is relatively low. In their audit of 1000 laparoscopic lymphadenectomies for gynaecologic cancers (including 182 endometrial carcinomas), Querleu et al. described a 2% rate of intraoperative complications with no associated lethality [42]. 71 symptomatic lymphocysts were observed and mostly managed by radiological drainage; 15% required surgery. In a recent study, Ghezzi et al. noticed that symptomatic lymphocysts were more frequent after laparotomy staging (15.4%) compared to laparoscopy (0.9%) [43], suggesting that some drawbacks of LND for EC might be dependent on the surgical approach and could be avoided by spreading minimal invasive techniques. 

Among the complications of LND, lower limb lymphedema remains the most significant concern. In Querleu's study, its rate was 1.5% but was probably underestimated due to the limited followup (6 months). Indeed, with a longer followup, Ghezzi et al. reported a rate of lymphedema of 14% and found no differences between laparoscopic and open surgery procedures. Todo et al. determined that adjuvant radiation therapy, removal of the circumflex iliac LN distal to the external iliac LN, and resection of more than 31 nodes were risk factors for the development of lower extremity lymphedema [44]. Finally, paraaortic lymphadenectomy is associated with a doubling in the risk of a 30-day morbidity [45, 46]. These findings emphasize the importance of careful patient selection for lymphadenectomy and underline the requirement of developing reliable and less invasive procedures as an alternative to standard LND.

In summary, LN metastasis is a real indicator of poor prognosis and requires an adapted adjuvant therapy. To date, lymphadenectomy is still the standard technique to assess the lymphatic spread of EC. However, its morbidity prompts us to define objective criteria to select patients who will benefit from such extensive staging.

## 4. Predictors of Lymph Node Metastases and Patients Stratification

Despite the debate on prognostic and therapeutic relevance of LND in early-stage EC, most of the investigators agree on stratifying patients into groups according to the risk of nodal involvement. Most of the risk factors for LNM are well known, histological type, tumor grade, lympho-vascular space involvement, and depth of myometrial invasion. GOG 33 reported that the overall incidence of LN involvement in clinical stage I EC rises from 3% in grade 1 to 9% in grade 2 and 18% in grade 3. 20% of stage IB patients had LN metastases, compared to less than 5% of stage IA [3]. The European Society for Medical Oncology (EMSO) subdivided early-stage EC patients into 3 risk categories for disease relapse and survival [47] as follows: (1) low risk (LR): stage IA, grade 1 or 2, type 1 neoplasm; (2) intermediate risk (IR): stage IB, grade 1 or 2, type 1 neoplasm/stage IA, grade 3, type 1 neoplasm; (3) high risk (HR): stage IB, grade 3, type 1 neoplasm/type 2 neoplasms.

**Figure 1:**
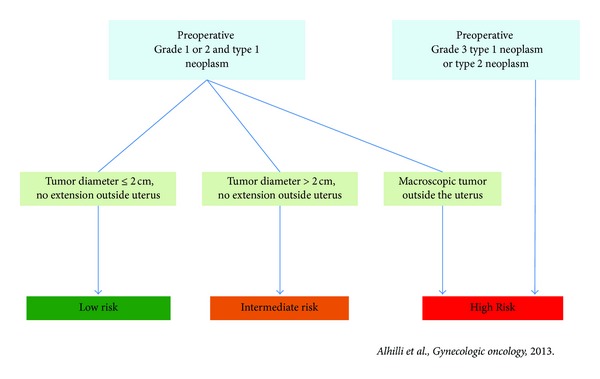
Stratification into risk categories.

In a recent retrospective study, Alhilli et al. [48] introduced a new risk stratification based on pre- and peroperative criteria including tumor size (Figure 1). LR patients had a very low risk of lymphatic dissemination or recurrence (<1%). Conversely, intermediate- and high-risk patients had a substantial risk of lymph node metastasis and recurrence (17%). 

Todo et al. described a preoperative scoring system based on tumor volume measured by MRI, serum CA125 level, tumor grade, and histological type [49]. This system was designed to predict both pelvic and paraaortic LN metastasis risks, according to cut-off values for each risk factor. LNM occurred in 3.3% in the LR group (no risk factor), 11.7% in the IR group (only 1 risk factor), and 36.7% in the HR group (2 risk factors). 

**Figure 2:**
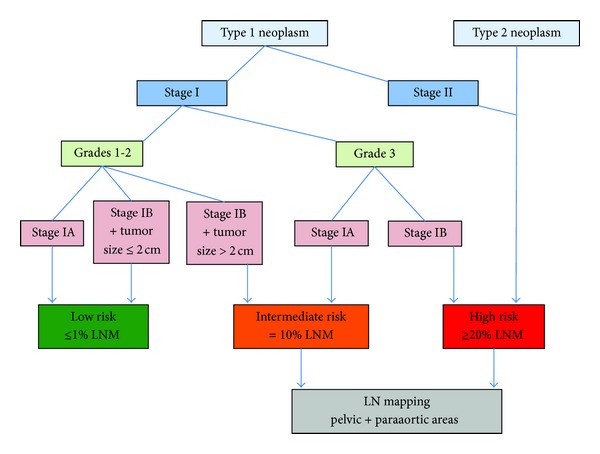
Indications of LN mapping integrating both Alhilli et al. and EMSO criteria (LNM: lymph node metastasis).

While all these classifications differ in their design and criteria, they demonstrate that there is a group of LR patients who should not undergo LND and that conversely LN sampling should be performed in IR and HR groups to determine optimal indications for adjuvant therapy (Figure 2). Since therapeutic relevance of LND is more than ever unclear and its morbidity is a real concern, less invasive procedures have been developed in the past 10 years. 

## 5. Sentinel Node Biopsy

The validity of the sentinel lymph node (SLN) concept has been demonstrated in melanoma, breast, and vulvar cancers [50–52]. The rationale of this procedure is to detect and remove selectively the first node(s) in a regional lymphatic basin that receives lymph flow from the primary tumour. Pathological status of these nodes may accurately predict the node status of the patient. Thus, when the sentinel node is negative, a complete lymphadenectomy can be avoided, resulting in reduced morbidity and optimized resources. Although SLN detection remains in a preliminary stage of evaluation in EC, there is an increasing interest in this technique. 

Whereas dual labelling method using both blue dye and radiocolloid (technetium^99 m^) has been shown to provide better results in terms of detection rate in comparison to blue dye alone [53], there is still no consensus about the most accurate method to identify SLN in patients presenting with EC. In 2011, Kang et al. performed a meta-analysis of 26 eligible studies and reported that the use of pericervical injection was correlated with increased detection rate, while hysteroscopic injection was associated with lower detection rate. Subserosal injection performed alone was correlated with decreased sensitivity [54]. Cervical injection has been criticized because it might not reflect the expected uterine lymphatic drainage resulting in a low detection rate in paraaortic area [55, 56]. Nevertheless, in a prospective multicentre study, Ballester et al. identified paraaortic SLN in 5% of the patients, using a cervical dual labeling method [33]. More recently and using a similar method, How et al. detected paraaortic SLN in 16% of their 100 patients [57]. Abu-Rustum et al. associated a combined intracervical injection with a blue dye subserosal injection leading to identify 3% of the SLN in the paraaortic area [58]. Several authors consider that the hysteroscopic injection represents the optimal method to highlight the complete lymphatic drainage of the uterus, but it remains clearly less applicable for physicians and might be difficult to accept for patients [56, 59]. 

Kang et al. computed overall detection rate and sensitivity of 78% and 93%, respectively [54]. The authors concluded that SLN biopsy had shown good performance that should be balanced with significant small study effects. In more recent studies, the detection rates were 92% (How et al.) and 88% (Ballester et al.). Bilateral detection rates were, however, much lower, 72% and 69%, respectively, and sensitivities were 89% and 84% (considering the patient as the unit). The false-negative rates in these studies were 11% and 16%, respectively. Furthermore, lymph node staging can be improved by the potential detection of micrometastases by the ultrastaging (serial section and immunohistochemistry) of a limited number of nodes. This may enhance the stratification of intermediate-risk patients and assist intailoring adjuvant therapy. 

SLN mapping may then provide an ideal midterm, reducing the unnecessary complete LND without understaging the patients. However, the relatively low detection rate and the nuclear medicine requirements represent obvious obstacles to the spread of such a technique. 

## 6. Peroperative Algorithm

Blue dye single labelling for SLN procedure is a very simple but largely unreliable method, hindered by low detection and high false-negative rates [62–64]. Nonetheless, its integration in a Peroperative Algorithm (POA) improves its effectiveness and it could be a substitute to complete LND in a high proportion of patients presenting with EC, without the need for nuclear medicine facilities [65, 66]. The steps of the POA, as described by Barlin et al. [67], are as follows: (1) peritoneal and serosal evaluations and washings; (2) retroperitoneal evaluation including excision of all mapped SLNs and suspicious nodes regardless of mapping; and (3) if there is no mapping on a hemipelvis, a side-specific pelvic, common iliac, and interiliac, lymph node dissection (LND) is performed. Paraaortic LND is performed at the attendings' discretion. The authors included 498 patients in a retrospectivly designed study. Using blue dye SLN procedure, detection rate was 81% and false-negative rate was 15%. After applying the algorithm, the false-negative rate dropped to 2% and sensitivity by definition was 100%. 

POA accuracy and simplicity make it a promising approach to assess LN status in EC patients. It might also provide an interesting cost effectiveness ratio. Nevertheless, its relevance should be confirmed in prospective trials.

## 7. Excluding LN Evaluation from Surgical Management of Early-Stage EC

MRC ASTEC and Panici et al. randomized trials specifically focused on the therapeutic value of pelvic LND and did not demonstrate any benefit from LN assessment in early-stage EC [10, 11]. However, the strength of their conclusions was hindered by several methodological limitations, resulting in a greater confusion surrounding the actual value of LND. Interestingly, other prospective studies might provide part of the answer. In PORTECs 1 and 2, staging LND was an exclusion criterion [68, 69]. PORTEC 1 aimed to compare the outcomes and adverse effects between pelvic external beam radiotherapy (EBRT) and surgery alone in patients presenting with early-stage EC, except from stage IB grade III that all received EBRT [68]. Indeed, patients in the control arm only underwent a total hysterectomy with bilateral salpingo-oophorectomy, without additional LND or adjuvant radiation therapy. Their overall 5-year survival was 85% (versus 81% in the radiotherapy group) and consistent with data from retrospective studies involving LND [2]. A 5-year locoregional recurrence rate was significantly higher compared to radiotherapy group (14% versus 4%, *P* < 0.001). Most relapses were restricted to the vagina (75%), while pelvic recurrences were only observed in 3.4% of the control group patients. That was consistent with Mayo Clinic retrospective data, involving LND for IR patients [48]. 

PORTEC 2 compared EBRT and vaginal brachytherapy in patients with intermediate- and high-risk EC [69]. Pelvic recurrence rates were low in both groups (0.6% for EBRT and 3.3% for VBT, *P* = 0.06), consistent with the radiotherapy group of prospective trial GOG 99 involving systematic pelvic and paraaortic LND (0.5%) [70].

These findings indirectly support the conclusions of MRC ASTEC and Panici et al. trials, showing no benefit from systematic LND in LR and HR subgroups. Nevertheless, it remains unclear if minimal LN assessment (SLN or POA procedures) could reduce morbidity through avoiding unnecessary postoperative radiotherapy. Furthermore, prospective data are still lacking for HR subgroup, since such patients in PORTEC 1 were excluded from randomization and all received adjuvant EBRT.

## 8. Conclusion

16 to 22% of clinical early-stage EC are upstaged after surgical procedure [3, 71]; subsequently many institutions recommend to perform a lymph node mapping in IR and HR stage I patients and in advanced diseases. Comprehensive surgical staging is, to date, the only accurate way to provide a proper evaluation of the lymphatic spread of the disease that has a clear impact on prognosis and adjuvant therapies. The interest of lymph node mapping should only be considered from this point of view, avoiding the pointless considerations about its supposed therapeutic virtue. The current approach for lymphatic assessment in EC should, thus, provide the best evaluation with the lowest morbidity. A systematic preoperative stratification should be performed to determine the patients that would benefit from LN mapping and might be in certain circumstances completed by a peroperative analysis, as proposed by Alhilli et al. [48]. As many authors, we believe that the lymphatic exploration should be restricted to IR and HR patients. Surgical staging should start with a low-invasive technique, such as SLN procedure or POA, that might be extended to complete LND in the cases of failed mapping. Indeed, complete lymphadenectomy should not be performed primary.

Altogether, the review of the literature provides solid ground for LN staging in patients with IR or HR. The less invasive modality should be preferred. Future studies might use molecular biology parameters that might define better HR and IR patients. LN staging will lead to the optimisation of adjuvant therapies.
